# Long Term Outcome of Postoperative Atrial Fibrillation After Cardiac Surgery—A Propensity Score-Matched Cohort Analysis

**DOI:** 10.3389/fcvm.2021.650147

**Published:** 2021-04-27

**Authors:** Jung-Chi Hsu, Chen-Yu Huang, Shu-Lin Chuang, Hsu-Yu Yu, Yih-Sharng Chen, Chih-Hsien Wang, Lian-Yu Lin

**Affiliations:** ^1^Division of Cardiology, Department of Internal Medicine, Camillian Saint Mary's Hospital Luodong, Yilan, Taiwan; ^2^Division of Cardiology, Department of Internal Medicine, National Taiwan University Hospital, Taipei, Taiwan; ^3^Division of Cardiology, Department of Internal Medicine, Taoyuan General Hospital, Ministry of Health and Welfare, Taoyuan, Taiwan; ^4^Department of Medical Research, National Taiwan University Hospital, Taipei, Taiwan; ^5^Graduate Institute of Epidemiology and Preventive Medicine, College of Public Health, National Taiwan University, Taipei, Taiwan; ^6^Division of Cardiovascular Surgery, Department of Surgery, National Taiwan University Hospital and College of Medicine, National Taiwan University, Taipei, Taiwan; ^7^Department of Internal Medicine, College of Medicine, National Taiwan University, Taipei, Taiwan; ^8^Division of Electrophysiology, Cardiovascular Center, National Taiwan University Hospital, Taipei, Taiwan

**Keywords:** postoperative atrial fibrillation, open heart surgery, coronary artery bypass grafting, stroke, atrial fibrillation

## Abstract

**Background:** Postoperative atrial fibrillation (POAF) results in a longer hospital stay and excess mortality. However, whether POAF would increase stroke rate has been debated for years. When and how long should anticoagulation be used to prevent stroke are unknown. In the study, we planned to investigate the clinical demographics and long-term outcomes of POAF after cardiac surgery in a single-center cohort.

**Methods:** The cohort study used a database from National Taiwan University Hospital, a single tertiary medical center in Taiwan, between 2007 and 2017, to identify patients with prior normal sinus rhythm developing POAF after cardiac surgery. Patients without POAF after cardiac surgery were used as controls. Propensity score matching with 1:1 ratio and Cox regression models were employed to estimate the risk of transient ischemic accident (TIA) or ischemic stroke.

**Results:** From 2007 to 2017, a total of 8,374 patients received open-heart surgery, in which 1,585 patients with a history of AF were excluded. The overall incidence of TIA/ischemic stroke was 3.9% in a median 9.2-years of follow-up. After propensity matching, 1,965 matched paired subjects were included for analysis. Postoperative atrial fibrillation was associated with an increased risk of future AF [Hazard ratio (HR) 1.40, 95% confidence interval (95%CI) = 1.09–1.79, *p* = 0.008] and heart failure (HF) hospitalization (HR 1.58, 95%CI 1.23–2.04, *p* < 0.001); however, POAF did not significantly correlate with the risk of TIA/ischemic stroke (HR 1.17, 95%CI 0.85–1.60, *p* = 0.043). Kaplan-Meier analysis showed that POAF was a significant predictor for future AF, HF hospitalization, and overall mortality, but not for TIA/ischemic stroke.

**Conclusion:** In the Asian population, POAF after cardiac surgery increased the risk of future AF, HF, and overall mortality, but was not associated with future TIA/ischemic stroke.

## Introduction

Atrial fibrillation (AF) is the most common perioperative arrhythmia, with an incidence of 3% in major non-cardiac surgery. The incidence was estimated to be 11–40% in patients receiving coronary artery bypass grafting (CABG) and even higher (>50%) after valvular surgery (1, 2). New-onset AF during the perioperative period could be provoked by physiological stress and sepsis and were considered to be associated with both short and long-term risks of stroke (3). Nevertheless, cardiac operations-associated AF, so-called Postoperative atrial fibrillation (POAF), appears to be a consequence of direct response of pericardial inflammation and is often believed to be self-limiting and probably has little impact on long-term outcome since the majority of patients return to normal sinus rhythm within a few weeks after surgery (4). Even though some studies have shown that new-onset POAF following CABG was associated with a significantly higher risk of short and long-term mortality, the long-term stroke risk remained controversial (5, 6). Some reported no association (7, 8), while some advocated increased risk of stroke after cardiac surgery (9–11).

Despite lack of strong evidence, the consensus of AHA/ACC/HRS guidelines recommended anticoagulation therapy for patients with prolonged duration of POAF (>48 h) and concomitant multiple stroke risk factors (12). The 2017 EACTS guidelines also recommended giving anticoagulation therapy to patients who developed POAF after cardiac surgery to avoid early stroke (13).

Since POAF is usually transient, when the sinus rhythm restores, whether the long-term stroke risk increases is still not known. In this study, we plan to investigate the long-term outcomes of the patients with POAF after cardiac surgery.

## Methods

### Data Collection

The study database was from National Taiwan University Hospital integrated Medical Database (NTUH-iMD) which was composed of detailed medical and procedural information from a tertiary medical center in Taiwan. Atrial fibrillation and its occurrence time were identified by the diagnosis of electronic health records and the electrocardiograms. Comorbidities including hypertension (HTN), diabetes mellitus (DM), hyperlipidemia, heart failure (HF), coronary artery disease (CAD), myocardial infarction (MI), chronic obstructive pulmonary disease (COPD), peripheral arterial occlusive disease (PAOD), chronic kidney disease (CKD) were also coded from the records. Prescription information were categorized into antiarrhythmic agents, beta-blockers, angiotensin-converting enzyme inhibitors (ACEI), angiotensin receptor blockers (ARB), mineralocorticoid-receptor antagonists (MRA), and anticoagulants including direct oral anticoagulants (DOAC) and warfarin. The open heart procedures included CABG, valvuloplasty, and valve replacement. The echocardiographic data and outcomes including TIA, ischemic stroke, HF hospitalization, and mortality were also obtained from the electronic health records. The study was approved by the Institutional Review Board (IRB) of National Taiwan University Hospital.

### Patient Selection

In this study, all patients above 18 years of age undergoing first-time open-heart surgery at the National Taiwan University Hospital from February 1, 2007, to February 30, 2017 were included for analysis. Patients with previous documented AF, who received anticoagulant therapy within 6 months before the open-heart surgery or during the follow-up period, could not survive the open-heart procedures, could not receive regular follow-up at the out-patient clinics were all excluded. The definition of POAF was new-onset AF, which sustained for over 30 s, detected by either continuous telemetry in the intensive care unit, standard 12-lead electrocardiogram, or implanted devices. Future AF was defined as documented AF 1 month after surgery or discharge. All medical records were followed until their last clinical visit, repeat cardiac surgery, or death. The index date of outcomes was defined as the date of diagnosis.

### Statistical Analysis

Continuous variables were denoted as mean (SD) while categorical variables were presented as numbers and percentages. Baseline demographics between groups were compared by using Student's *t*-test for continuous variables and Chi-square test for categorical variables.

Missing data for variables of interest were handled by multiple imputations with chained equations before propensity score matching. Propensity score matching was used to adjust for potentially confounding variables. We calculated the propensity scores by hierarchical logistic regression. Covariates included in the logistic model were age, gender, HTN, DM, hyperlipidemia, COPD, CHF, CAD, CKD, CHA2DS2-VASc score, echocardiography parameters, and medications. Considering the statistical power and distribution of sample size, patients with POAF were matched 1:1 to non-POAF patients, using the nearest neighbor method without replacement and with caliper 0.2 which was statically optimal width (14, 15). The matching result was estimated by calculating the absolute standard differences of baseline characteristics between patients with POAF and non-POAF. A value of <10% was considered well-matched. A Cox proportional hazard model was used to estimate the association between POAF and outcomes. Results were expressed as hazard ratios (HRs) with 95% confidence intervals (CIs). Factors that were statistically significant in univariate analysis would be put into multivariate analysis. Survival analyses were illustrated by the Kaplan-Meier curve. A two-tailed *p*-value of <0.05 was considered statistically significant. All statistical analyses were performed using SPSS V.25.0 (SPSS, Inc., Chicago, Illinois) and R software (R software version 3.3.3).

## Results

### Baseline Characteristics

A total of 8,374 patients received cardiac surgery between 2007 and 2017, of which 1,585 patients were excluded because of pre-existing AF. After excluding patients with anticoagulant, a total of 6,267 patients was enrolled to this cohort. As shown in Table 1, before propensity matching, patients with POAF were elder (65.2 ± 13.6 vs. 60.5 ± 13.5, *p* < 0.001), more often female (25.5 vs. 22.0%, *p* = 0.002), had fewer comorbidities including HTN (66.9 vs. 69.6%, *p* = 0.035), hyperlipidemia (44.0 vs. 62.0%, *p* < 0.001), and CAD (81.1 vs. 87.7%, *p* < 0.001). The prevalence of other comorbidities such as COPD (10.2 vs. 8.8%, *p* = 0.037), CHF (26.8 vs. 14.3%, *p* < 0.001), CKD (26.9 vs. 17.5%, *p* < 0.001), together with the average CHA2DS2-VASc score (2.64 ± 1.60 vs. 2.15 ± 1.49, *p* < 0.001) were significantly higher in POAF group. The echocardiographic parameters including left ventricular ejection fraction (LVEF) (54.00 ± 12.38 vs. 59.62 ± 12.38, *p* < 0.001) was lower while the left ventricular mass (LVM) (231.94 ± 84.13 vs. 209.50 ± 70.96, *p* < 0.001) and the left atrial size (3.89 ± 0.73 vs. 3.64 ± 0.67 mm, *p* < 0.001) were larger in patients with POAF. The chances of taking MRA (11.3 vs. 6.8%, *p* < 0.001) and anti-arrhythmic agents (1.0 vs. 0.4%, *p* < 0.001) were higher in patients with POAF while less patients in the POAF group taking beta-blockers (44.8 vs. 62.2%, *p* < 0.001) and ACEI/ARBs (ACEIs 6.0 vs. 9.1%, *p* < 0.001; ARBs 30.7 vs. 41.0%, *p* < 0.001). After propensity matching, 1,965 paired matched patients were included for analysis. As demonstrated in Table 1, all of the absolute standard differences of the demographic and clinical characteristics between groups were within 10% and the *p*-value of the overall balance test (Hansen&Bowers) was 0.962, which was suggestive of well-matched. After PS matching, only echocardiographic parameters remained significantly different between groups. Multiple regression would be further applied to adjust all these confounders.

**Table 1:** Basic characteristics of enrolled patients before and after propensity score matching.

	**Pre-Match**				**Post-Match**			
	**POAF** **(*N* = 2,015)**	**Non-POAF** **(*N* = 4,252)**	**Std. Diff**.	***p*-Value**	**POAF** **(*N* = 1,965)**	**Non-POAF** **(*N* = 1,965)**	**Std. Diff**.	***p*-Value**
Age	65.2 ± 13.6	60.5 ± 13.5	0.35	**<0.001**	65.0 ± 13.6	64.5 ± 13.8	0.03	0.339
Gender (male)	1,502 (74.5%)	3,318 (78.0%)	−0.08	**0.002**	1,469 (74.8%)	1,501 (73.4%)	−0.04	0.235
Hypertension	1,349 (66.9%)	2,959 (69.6%)	−0.06	0.035	1,319 (67.1%)	1,349 (67.7%)	−0.03	0.305
Type 2 DM	891 (44.2%)	1,854 (43.6%)	0.01	0.647	871 (44.3%)	894 (45.5%)	0.02	0.461
Hyperlipidemia	886 (44.0%)	2,636 (62.0%)	−0.36	**<0.001**	878 (44.6%)	903 (46.0%)	−0.03	0.423
COPD	205 (10.2%)	375 (8.8%)	0.08	**0.037**	199 (10.1%)	210 (10.7%)	−0.02	0.566
CHF	541 (26.8%)	608 (14.3%)	0.28	**<0.001**	512 (26.1%)	465 (23.7%)	0.05	0.083
CAD	1,635 (81.1%)	3,731 (87.7%)	−0.17	**<0.001**	1,607 (81.8%)	1,616 (82.2%)	−0.01	0.709
CKD	542 (26.9%)	744 (17.5%)	0.19	**<0.001**	500 (25.4%)	496 (25.2%)	0.01	0.883
CHA2DS2-VASc score	2.64 ± 1.60	2.15 ± 1.49	0.31	**<0.001**	2.61 ± 1.59	2.56 ± 1.59	0.03	0.321
UCG								
LVEF (%)	54.00 ± 12.38	59.62 ± 12.38	−0.11	**<0.001**	43.23 ± 13.05	48.42 ± 12.17	−0.03	**<0.001**
LV mass (g)	231.94 ± 84.13	209.50 ± 70.96	−0.11	**<0.001**	230.33 ± 82.02	217.48 ± 77.94	−0.03	**<0.001**
LA size (mm)	3.89 ± 0.73	3.64 ± 0.67	−0.08	**<0.001**	3.88 ± 0.73	3.73 ± 0.72	−0.02	**<0.001**
E	89.69 ± 34.61	81.37 ± 29.53	−0.07	**<0.001**	89.17 ± 34.33	85.61 ± 31.97	−0.02	**0.001**
A	85.27 ± 29.67	81.37 ± 29.53	0.02	0.502	85.45 ± 29.78	86.02 ± 26.50	0.02	0.636
E/A	1.14 ± 0.62	1.01 ± 0.50	0.02	**<0.001**	1.13 ± 0.62	1.06 ± 0.57	0.02	**0.003**
DT	0.27 ± 2.44	0.26 ± 2.93	−0.04	0.934	0.27 ± 2.48	0.32 ± 4.27	0.01	0.711
Medication								
Anti-arrhythmic agents	20 (1.0%)	16 (0.4%)	0.06	**0.003**	15 (0.8%)	13 (0.7%)	0.01	0.704
Beta-blocker	903 (44.8%)	2,646 (62.2%)	−0.35	**<0.001**	893 (45.4%)	933 (47.5%)	−0.04	0.201
ACEI	121 (6.0%)	385 (9.1%)	−0.13	**<0.001**	120 (6.1%)	123 (6.3%)	−0.01	0.843
ARB	618 (30.7%)	1,744 (41.0%)	−0.22	**<0.001**	611 (31.1%)	637 (32.4%)	−0.03	0.373
MRA	228 (11.3%)	290 (6.8%)	0.14	**<0.001**	214 (10.9%)	211 (10.7%)	0.05	0.878
Outcome								
Future AF	267 (13.1%)	343 (8.1%)		**<0.001**	261(13.3%)	169 (8.6%)		**<0.001**
TIA/Stroke	82 (4.1%)	164 (3.9%)		0.686	77 (3.9%)	75 (3.8%)		0.869
HF hospitalization	146 (7.3%)	208 (4.9%)		**<0.001**	144 (7.3%)	103 (5.2%)		**0.007**
Mortality	19 (0.9%)	9 (0.2%)		**<0.001**	18 (0.9%)	5 (032%)		**0.010**

*Absolute standard differences are defined as the difference in means, proportions, or ranks divided by the mutual standard deviation. DM, diabetes mellitus; COPD, chronic obstructive pulmonary disease; CHF, congestive heart failure; CAD, coronary artery disease; CKD, chronic kidney disease; TIA, transient ischemia accident; LVEF, left ventricular ejection fraction; LV mass, left ventricular mass; LA size, left atrium size; ACEI, angiotensin-converting enzyme inhibitors; ARB, angiotensin receptor blockers; MRA, mineralocorticoid-receptor antagonists; AF, atrial fibrillation; HF hospitalization, heart failure hospitalization*.

### Outcomes

**Figure 1:**
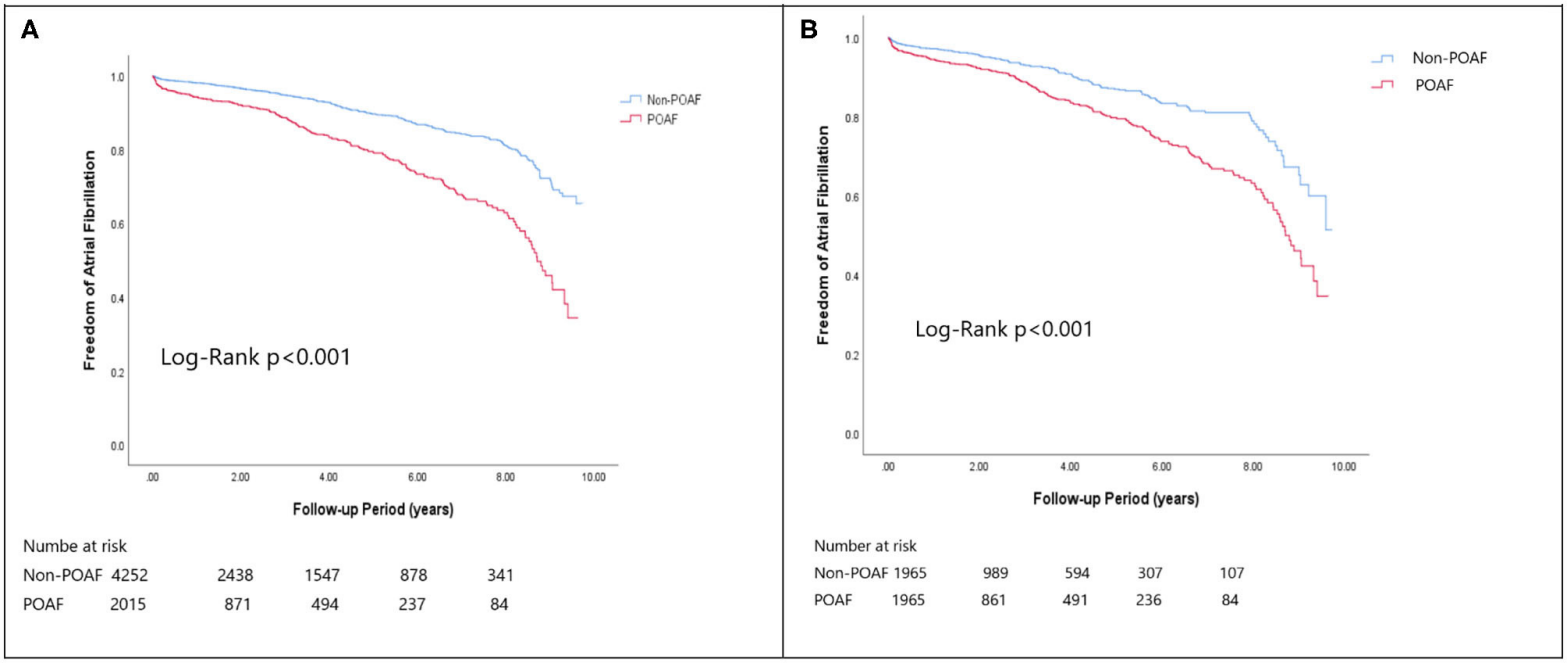
The Kaplan-Meier curve of **(A)** atrial fibrillation (crude) and **(B)** atrial fibrillation (matched).

**Figure 2:**
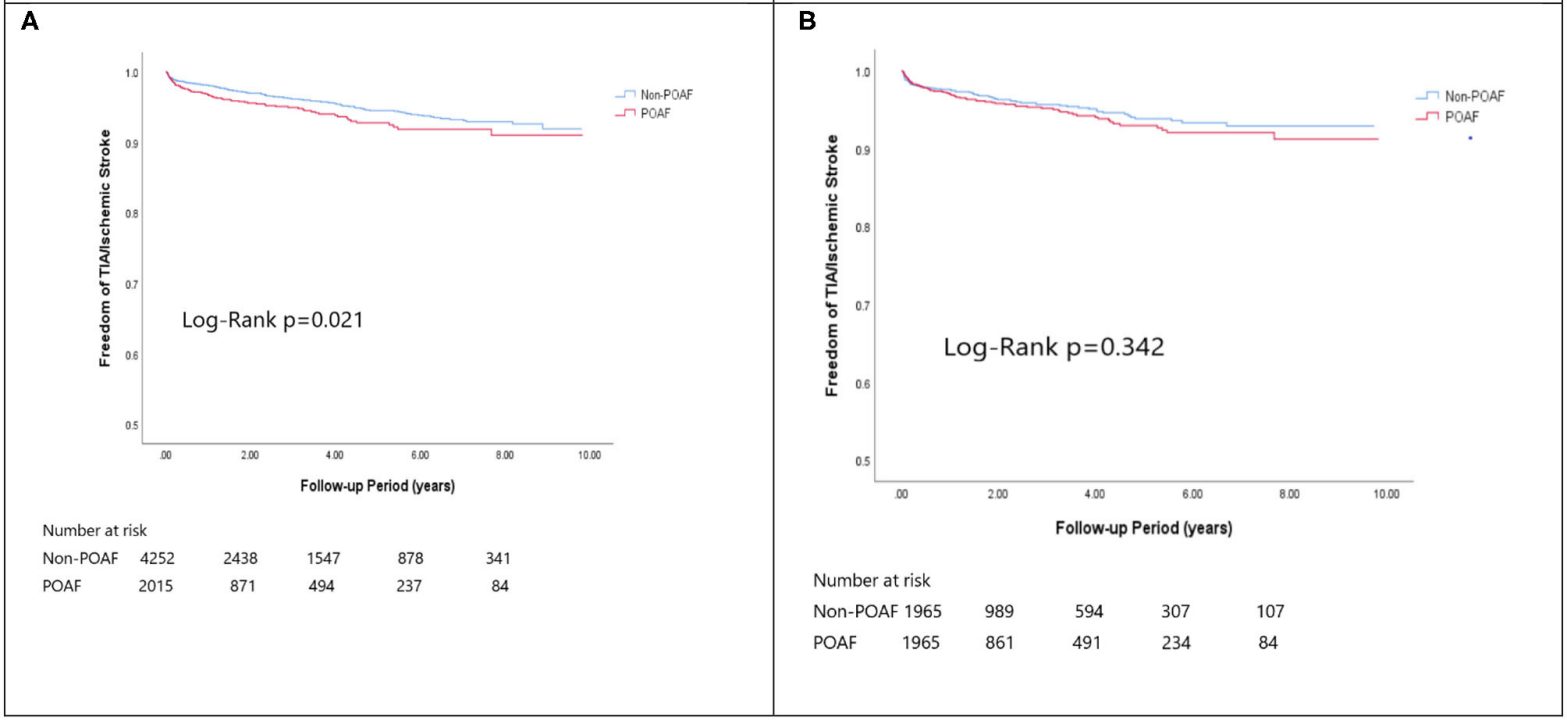
The Kaplan-Meier curve of **(A)** TIA/Ischemic stroke (crude) and **(B)** The Kaplan-Meier curve of atrial fibrillation (matched).

**Figure 3:**
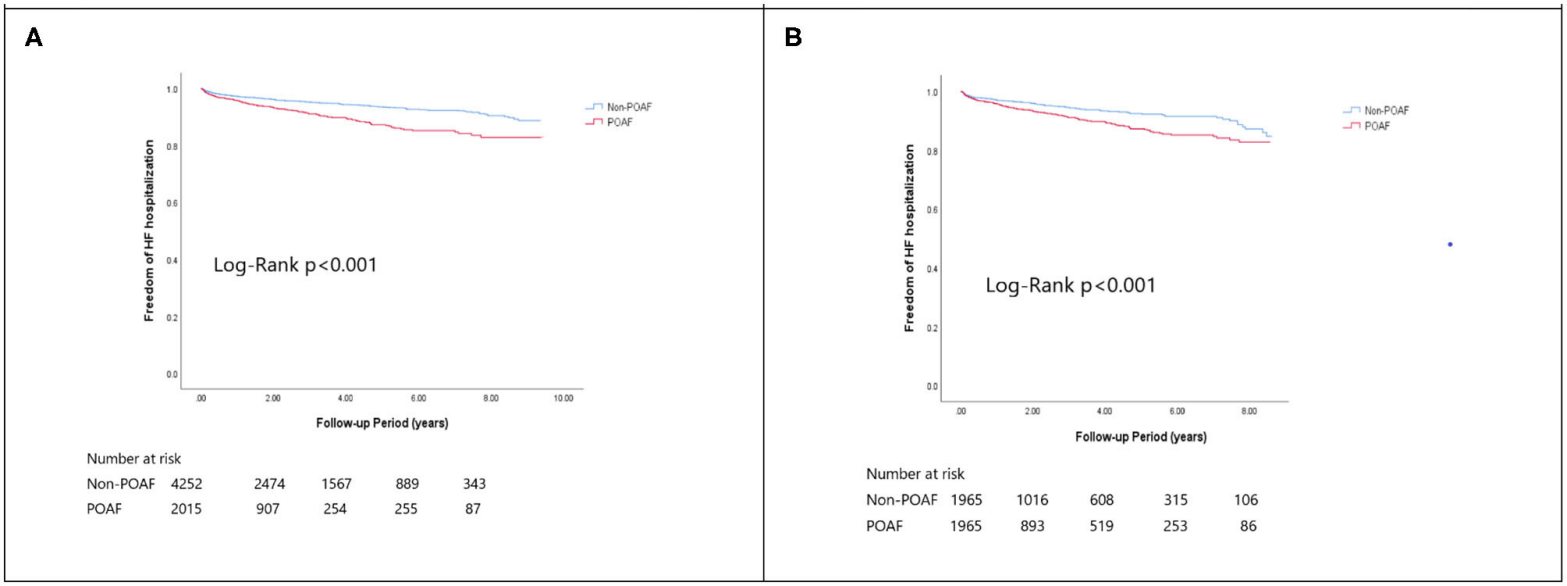
The Kaplan-Meier curve of **(A)** HF hospitalization (crude) and **(B)** HF hospitalization (matched).

**Figure 4:**
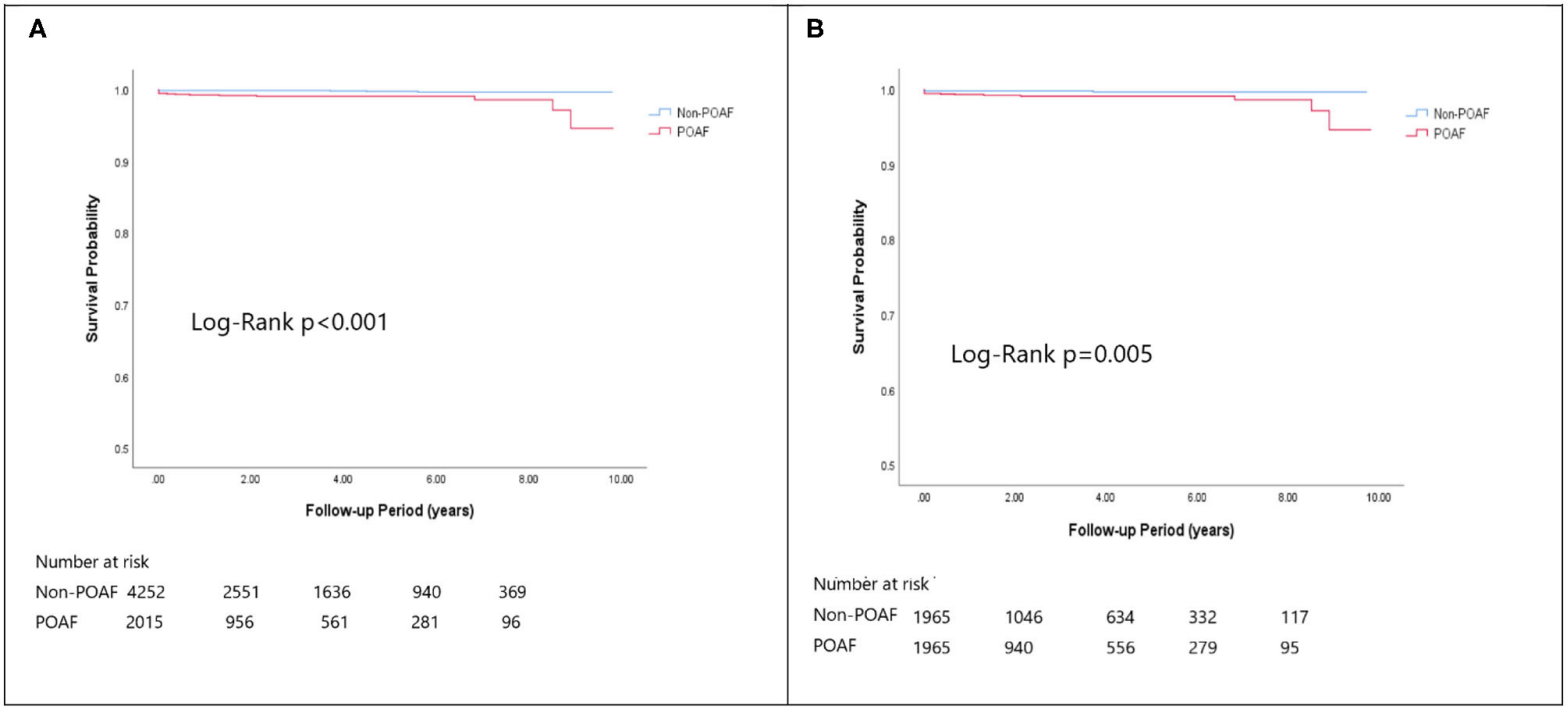
The Kaplan-Meier curve of **(A)** overall mortality (crude) and **(B)** overall mortality (matched).

In the matched cohort, the overall TIA/ischemic stroke rate was 3.9% in the POAF group and 3.8% in the control group during a median of 9.2-year follow-up. The incidence of future AF was higher in patients with POAF (13.1 vs. 8.1%, *p* < 0.001). Other outcomes including the rates of CHF hospitalization (7.3 vs. 4.9%, *p* < 0.001), and overall mortality (0.9 vs. 0.2%, *p* < 0.001) were significantly higher in patients with POAF. Univariate cox regression analysis revealed that POAF, CHA2DS2-VASc score, COPD, CKD, poorer LVEF, larger LA size were significant predictors for future AF. Multivariate analysis showed that POAF (HR 1.33, 95%CI 1.04–1.71, *p* = 0.025), CHA2DS2-VASc score (HR 1.18, 95%CI 1.18–1.39, *p* < 0.001), CKD (HR 2.08, 95%CI 1.60–2.69), poorer LVEF (HR 0.98, 95%CI 0.97–0.99, *p* < 0.001), and larger LA size (HR 1.34, 95%CI 1.14–1.57, *p* = 0.001) were predictors for future AF (Table 2). For TIA/ischemic stroke, univariate, and multivariate analysis showed that CHA2DS2-VASc score (HR 1.63, 95%CI 1.42–1.87), hyperlipidemia (HR 1.68, 95%CI 1.08–2.61), CKD (HR 1.56, 95%CI 1.01–2.40), and higher LV mass were significantly associated with TIA/ischemic stroke (HR 1.003, 95%CI 1.000–1.006, *p* = 0.027). Of noted, POAF was not significantly associated with future TIA/ischemic stroke (HR 1.17, 95%CI 0.85–1.60, *p* = 0.343). For HF hospitalization, univariate analysis showed that POAF (HR 1.58, 95%CI 1.23–2.04, *p* < 0.001), CHA2DS2-VASc score (HR 1.14, 95%CI 1.06–1.24), COPD (HR 1.52, 95%CI 0.97–2.37), CKD (HR 3.08, 95%CI 2.39–3.95), poorer LVEF (HR 0.98, 95%CI 0.96–0.99, *p* < 0.001), and higher LV mass (HR 1.002, 95%CI 1.000–1.004, *p* = 0.039) were associated with HF hospitalization, in which POAF (HR 1.82, 95%CI 1.29–2.56, *p* = 0.001), CKD (HR 2.76, 95%CI 1.94–3.91), and poorer LVEF (HR 0.98, 95%CI 0.97–0.99, *p* < 0.001) were predictors in multivariate analysis. As for overall mortality, univariate analysis revealed that POAF (HR 3.73, 95%CI 1.39–10.06, *p* = 0.009), CKD (HR 3.29, 95%CI 1.45–7.46), and poorer LVEF (HR 0.96, 95%CI 0.93–0.99, *p* = 0.008) were significant predictors, but after adjustment, only CKD (HR 2.95, 95%CI 1.14–7.65) and poorer LVEF (HR 0.967, 95%CI 0.939–0.996, *p* = 0.025) remained significant. Kaplan-Meier analysis showed that the chance of future AF was significantly higher in patients with POAF both before (Log-rank *p* < 0.001) (Figure 1A) and after (Figure 1B) propensity matching (Log-rank *p* < 0.001), while the risk of future TIA/ischemic stroke was significantly higher in patients with POAF before matching (Log-rank *p* = 0.021) (Figure 2A) but became non-significant after matching (Log-rank *p* = 0.342) (Figure 2B). Kaplan-Meier analysis also demonstrated that patients with POAF had higher chances of HF hospitalization and mortality both before and after matching (all Log-rank *p* < 0.001) (Figures 3, 4).

**Table 2:** Univariate and multivariate Cox regression models in patients with postoperative atrial fibrillation in matched cohort.

	**Future atrial fibrillation**				**TIA/Ischemic stroke**				**Heart failure hospitalization**				**Mortality**			
	**Univariate**		**Multivariate**		**Univariate**		**Multivariate**		**Univariate**		**Multivariate**		**Univariate**		**Multivariate**	
**Parameters**	**HR** ** (95%CI)**	***p*-Value**	**HR** ** (95%CI)**	***p*-Value**	**HR** ** (95%CI)**	***p*-Value**	**HR** ** (95%CI)**	***p*-Value**	**HR** ** (95%CI)**	***p*-Value**	**HR** ** (95%CI)**	***p*-Value**	**HR** ** (95%CI)**	***p*-Value**	**HR** ** (95%CI)**	***p*-Value**
POAF	1.792 (1.476–2.176)	**<0.001**	1.401 (1.092–1.798)	**0.008**	1.166 (0.849–1.603)	0.343			1.581 (1.227–2.036)	**<0.001**	1.819 (1.292–2.563)	**0.001**	3.732 (1.385–10.055)	**0.009**	2.851 (0.923–8.806)	0.069
CHA2DS2-VASc score	1.419 (1.336–1.508)	**<0.001**	1.182 (1.182–1.397)	**<0.001**	1.651 (1.498–1.819)	**<0.001**	1.630 (1.422–1.867)	**<0.001**	1.143 (1.056–1.238)	**<0.001**	0.944 (0.843–1.058)	0.324	0.909 (0.691–1.196)	0.497		
Hyperlipidemia	0.936 (0.772–1.134)	0.936			1.443 (1.038–2.007)	**0.029**	1.675 (1.077–2.605)	**0.022**	0.994 (0.734–1.214)	0.651			0.767 (0.334–1.764)	0.532		
COPD	1.848 (1.451–2.354)	**<0.001**	1.067 (0.750–1.519)	0.719	1.226 (0.766–1.963)	0.395			1.647 (1.186–2.289)	**0.003**	1.515 (0.968–2.370)	0.069	0.793 (0.186–3.383)	0.754		
CKD	2.481 (2.048–3.005)	**<0.001**	2.076 (1.602–2.689)	**<0.001**	2.400 (1.741–3.309)	**<0.001**	1.557 (1.009–2.403)	**0.046**	3.078 (2.395–3.946)	**<0.001**	2.756 (1.940–3.914)	**<0.001**	3.290 (1.451–7.460)	**0.004**	2.950 (1.138–7.649)	**0.026**
LVEF	0.982 (0.974–0.990)	**<0.001**	0.987 (0.978–0.995)	**0.002**	0.994 (0.980–1.009)	0.441			0.975 (0.964–0.985)	**<0.001**	0.980 (0.969–0.991)	**<0.001**	0.962 (0.934–0.990)	**0.008**	0.967 (0.939–0.996)	**0.025**
LV mass	1.001 (0.999–1.002)	0.280			1.002 (1.000–1.005)	**0.036**	1.003 (1.000–1.005)	**0.019**	1.002 (1.000–1.004)	**0.032**	1.000 (0.998–1.002)	0.966	0.998 (0.992–1.005)	0.582		
LA size	1.335 (1.137–1.568)	**<0.001**	1.164 (0.980–1.382)	0.083	0.984 (0.737–1.315)	0.915			1.076 (0.857–1.351)	0.528			0.477 (0.223–1.024)	0.057		
Beta-blocker use	0.979 (0.806–1.190)	0.832			0.814 (0.591–1.120)	0.814			1.088 (0.842–1.405)	0.518			0.569 (0.242–1.336)	0.195		
ACEI/ARB use	0.943 (0.778–1.141)	0.545			1.133 (0.822–1.561)	0.446			0.877 (0.681–1.128)	0.877			0.579 (0.234–1.434)	0.238		

*HR, hazard ratio, with 95% confidence interval (CI), p < 0.05 statistically significant*.

*COPD, chronic obstructive pulmonary disease; CKD, chronic kidney disease; LVEF, left ventricular ejection fraction; LV mass, left ventricular mass; LA size, left atrium size; ACEI, angiotensin-converting enzyme inhibitors; ARB, angiotensin receptor blockers*.

## Discussion

In this study, we investigated the development of future AF and TIA/ischemic stroke after cardiac surgery during a long-term follow-up. We found that POAF increased the risk of future AF, HF hospitalization, and overall mortality, but was not associated with TIA/ischemic stroke.

In a large cohort study, the rate of new-onset perioperative AF during index hospitalization was 1.43% and the cumulative 1-year ischemic stroke rate was 1.47% after non-cardiac surgery and 0.99% after cardiac surgery. Perioperative AF was found to be significantly correlated with ischemic stroke, with a HR of 2.0 and 1.3 in non-cardiac and cardiac surgery, respectively (3). A very recent study reported a significantly increased risk of stroke, MI, and death at 1 year in patients with POAF following non-cardiac surgery (16). In cardiac surgery, another large cohort study revealed that the incidence of post-CABG AF was around 30%, which was much higher than non-cardiac surgery and the incidence varied little over time (9, 17–19). Post-CABG AF occurs most commonly 24–72 h after surgery, with a peak incidence on the second postoperative day. The onset is typically within 6 days after operation with 15–20% of episodes spontaneously converting to sinus rhythm within several hours (19, 20). It seems that although POAF is easier to develop in cardiac surgery but it carries a higher risk of stroke in non-cardiac surgery. One reason leading to this discrepancy might be attributed to different underlying pathophysiologies. For cardiac surgery, POAF tends to be triggered by direct irritation of the atrial tissues while in non-cardiac surgery, intrinsic atriopathy might be the leading cause and thus result in higher stroke risk. As a whole, the mechanisms of POAF are complex, including atrial ischemia, inflammation, hypoxia, acidosis, oxidative stress, electrolyte abnormalities, and intra-atrial conduction delay and dispersion of refractoriness of the atrial tissue (21). Interestingly, a small study showed that pulmonary vein isolation does not decrease the incidence of POAF or its clinical impact, suggestive of non-pulmonary vein mechanisms (22). In our study, the incidence of POAF was 32.1% which was compatible with previous studies.

Our study showed that patients with POAF were elder, had a higher prevalence of CKD, higher CHA2DS2-VASc score, and poorer echocardiographic profile, all of which are compatible with previous studies (23–26). Also noted, patients in POAF group were less frequently taking ACEI/ARBs and beta-blockers, both medications were proved to be able to prevent the development of POAF (27, 28).

Patients with secondary AF following clinical events such as surgery, infection, acute MI, or thyrotoxicosis, were found to carry higher long-term risks of stroke and mortality (19). Postoperative atrial fibrillation is a secondary AF, but it should be considered as an independent entity. Previous studies have shown that patients with POAF have taken a 3- to 8-time risk of developing future AF and have higher risks of cerebrovascular events and cardiovascular mortality (10, 11). However a cohort study demonstrated that AF developing late after operation, rather than POAF, is independently associated with long-term mortality (29). Another study also showed that patients with new-onset POAF after CABG had a lower long-term thromboembolic risk than those who had non-valvular AF (30). These findings are in accord with ours that POAF is not a predictor for future TIA/ischemic stroke. Since most studies focused on the early risk of ischemic stroke after cardiac surgery, our study is unique because it provided the first Asian long-term and large sample cohort data.

Given that POAF might not be associated with long-term prognosis, patients with POAF might be prone to late AF. Nevertheless, the long-term data for late AF in POAF patients remains sparse. Some studies have reported that one- to two-thirds of patients had late AF after CABG when closely monitoring (31–33). The incidence of late AF in our study was 15.9%, which was much lower than previous reports. The reasons are not unclear but might probably be related to differences in patients' characteristics or AF detection methods. The current guideline recommends anticoagulation therapy for patients with POAF for at least 4 weeks, but when to discontinue anticoagulants is still uncertain. Since the CHA2DS2-VASc score itself represented the risk factors for AF incidence, the mean CHA2DS2-VASc score in our patients receiving open heart surgery was above score 2 in both POAF and non-POAF group, suggesting that all patients should be aware of AF occurrence. Furthermore, the average of CHA2DS2-VASc score was 2.6 in our POAF group, which reached the criteria for anticoagulation therapy once late AF developed. As a result, for patients with POAF who are at high risk for late AF, physicians should carefully monitor those patients to start anticoagulation therapy at the right time.

## Limitation

The study has some limitations. First, this is a retrospective analysis from an integrated database from a single medical tertiary center in Taiwan. Second, AF was identified by diagnostic code, electrocardiogram, and medical history, not by long-term recorder, some patients with AF might be underdiagnosed.

## Conclusion

In conclusion, in this large Asian cohort, POAF after cardiac surgery increased the risk of future AF, HF, and overall mortality, but was not associated with future TIA/ischemic stroke.

## Data Availability Statement

The datasets presented in this article are not readily available. Requests to access the datasets should be directed to cactus146@gmail.com.

## Ethics Statement

The study was approved by the Institutional Review Board (IRB) of National Taiwan University Hospital.

## Author Contributions

L-YL, contributed to the conception, design, acquisition of the work, and critically revised the manuscript. C-YH, J-CH, and S-LC contributed to the analysis, or interpretation of data for the work. J-CH drafted the manuscript. All authors gave final approval and agree to be accountable for all aspects of work ensuring integrity and accuracy.

## Conflict of Interest

The authors declare that the research was conducted in the absence of any commercial or financial relationships that could be construed as a potential conflict of interest.
